# Waning Measles Immunity and Sustained Rubella Seroprotection Among Adults in Istanbul, Türkiye

**DOI:** 10.3390/medicina62091709

**Published:** 2026-09-06

**Authors:** Kutay Sarsar, Ismail Aytaç Acar, Sevim Meşe, Ali Ağaçfidan, Muammer Osman Köksal

**Affiliations:** Department of Medical Microbiology, Istanbul University Faculty of Medicine, 34093 Istanbul, Türkiye; kutay.sarsar@istanbul.edu.tr (K.S.); aytacacar55@gmail.com (I.A.A.); smese@istanbul.edu.tr (S.M.); ali.agacfidan@istanbul.edu.tr (A.A.)

**Keywords:** measles, rubella, mumps, MMR vaccine, seroepidemiology, seroprevalence, adult immunity, immunity gaps, ELISA, Türkiye

## Abstract

*Background and Objectives*: Despite long-standing MMR vaccination programmes, outbreaks of vaccine-preventable diseases continue to occur worldwide, raising concerns regarding waning immunity and susceptibility gaps in adult populations. Between 2017 and 2019 alone, WHO reported over 880,000 measles cases globally—a fourfold increase over the preceding two years—with outbreaks in both low- and high-income countries despite high childhood vaccination coverage. This study evaluated the seroepidemiology of measles, mumps, and rubella among adults in Türkiye over a nine-year period. *Materials and Methods*: A retrospective laboratory-based study was conducted using ELISA IgM and IgG results obtained between 2014 and 2022 at a tertiary-care university hospital. Seropositivity rates were analysed by year, age group, and sex. Temporal trends were assessed using the Cochran–Armitage trend test; factors associated with IgG seropositivity were assessed using multivariable logistic regression. *Results*: Among 10,407 adult test records, overall IgG seropositivity was 75.8% for measles, 94.9% for rubella, and 86.3% for mumps; IgM positivity remained low throughout (0.9%, 0.4%, and 1.9%, respectively). Measles IgG declined significantly from 78.4% in 2014–2019 to 62.6% in 2022 (*p* < 0.001), whereas Rubella IgG remained stable (93.2–95.7%) and Mumps IgG showed no significant temporal change. Seropositivity increased markedly with age for measles (18–29 years: 70.2% vs. ≥50 years: 93.4%; *p* < 0.001) and mumps (*p* = 0.030). Multivariable analysis confirmed that Measles IgG seropositivity was substantially lower in 2022 than in the pooled 2014–2019 period (OR = 0.432, 95% CI: 0.319–0.584, *p* < 0.001). Rubella seroprotection among women of reproductive age was 94.7% (95% CI: 93.9–95.5%), close to but not statistically distinguishable from the 95% population immunity benchmark used in rubella elimination frameworks. *Conclusions*: A significant measles immunity gap exists among younger adults in Türkiye, with Measles IgG seropositivity declining from 78.4% in 2014–2019 to 62.6% in 2022, consistent with a cohort effect whereby vaccine-era cohorts exhibit lower protection than older adults with naturally acquired immunity. Rubella immunity, by contrast, remained close to the 95% elimination benchmark throughout. These findings underscore the importance of targeted serological surveillance and evidence-based booster vaccination strategies.

## 1. Introduction

Measles, mumps, and rubella remain among the most important vaccine-preventable viral diseases despite widespread vaccination. The introduction of the combined measles–mumps–rubella (MMR) vaccine has dramatically reduced disease incidence, associated complications, and mortality, contributing substantially to global disease-control and elimination efforts: global measles deaths fell from an estimated 780,000 in 2000 to approximately 95,000 in 2024, an 88% reduction attributed to sustained vaccination coverage. Nevertheless, outbreaks continue even in countries with established programs, reflecting persistent immunity gaps and fragile herd protection as population immunity declines [1,2,3,4,5,6,7].

Among these infections, measles represents the greatest public health challenge because of its extraordinary transmissibility. With a basic reproduction number (R_0_) estimated between 12 and 18, measles requires population immunity levels exceeding 95% to interrupt transmission effectively [1,3,4]; a population-level benchmark distinct from the assay-based seroprevalence estimates reported later in this study, which describe a tested clinical sample rather than the general population. Although global measles mortality has declined markedly since vaccine introduction, recent years have seen a resurgence of cases in multiple regions, attributed to declining vaccine uptake, itself driven by vaccine hesitancy, misinformation, and reduced access to primary healthcare services [2,6,8], alongside disruptions in routine immunization services and the accumulation of susceptible individuals within communities. While COVID-19-related disruptions created immunity gaps in younger pediatric cohorts, gradual waning of vaccine-induced immunity over decades has concurrently left cohorts vaccinated in childhood, now adults, as a growing pool of susceptible young adults [9,10,11]; international travel and migration have further accelerated cross-border transmission into communities whose immunity had previously been sufficient to interrupt endemic circulation [6,12,13]. Mathematical modelling suggests that even modest reductions in vaccination coverage can lead to substantially more measles cases and severe complications over time [14]. Beyond direct protection against measles infection, vaccination has also been proposed to have non-specific beneficial effects on all-cause childhood mortality, although the evidence for this hypothesis is mixed. Earlier observational analyses reported reductions of up to 30–50% in all-cause childhood mortality attributable to measles-containing vaccination beyond its disease-specific effects, and a 2016 systematic review concluded that such vaccination was associated with a greater-than-expected mortality reduction, albeit based mainly on observational data of variable quality [15]; a more recent systematic review and meta-analysis of standard-titre measles vaccines, however, found no support for this non-specific-effects hypothesis, reporting non-significant pooled relative risks (RR = 1.00 and RR = 0.90) for all-cause mortality [16].

Rubella remains a major public health concern because of its teratogenic potential. Maternal infection during early pregnancy may result in congenital rubella syndrome (CRS), which is associated with severe fetal abnormalities, including congenital heart disease, cataracts, deafness, and neurodevelopmental impairment [7,17]. Rubella control strategies therefore focus on both reducing virus circulation and maintaining high immunity among women of reproductive age, since sustained population immunity is essential to achieving and preserving international rubella elimination targets [18].

Mumps presents a different epidemiological challenge. Although vaccination has dramatically reduced disease incidence, outbreaks continue to occur in highly vaccinated populations, particularly among adolescents and young adults. Increasing evidence links waning vaccine-induced immunity to the re-emergence of mumps outbreaks, compounded by differences between vaccine strains and circulating viruses [6,19,20]; antibody levels can decline years after vaccination, and while booster doses raise serological responses, their durability remains under investigation [20,21]. Monitoring population immunity has therefore become increasingly important in the post-elimination era: vaccination-coverage data show uptake but not immune protection, whereas seroepidemiological studies directly assess humoral immunity, identify susceptible populations, evaluate programme effectiveness, and detect immunity gaps that routine surveillance alone may miss [22,23]. Such studies are particularly important for highly contagious infections such as measles, where relatively small declines in immunity can substantially increase outbreak risk [14,22]. Serological investigations across countries reveal heterogeneous immunity profiles by age: younger adults relying predominantly on vaccine-induced immunity often show lower antibody prevalence than older cohorts who acquired immunity through natural infection before widespread vaccination, an effect attributed partly to ongoing immune boosting from exposure to circulating wild-type virus in older cohorts [22,23,24]. This raises concerns about the long-term persistence of vaccine-derived immunity and the potential accumulation of susceptible individuals within populations that otherwise appear adequately vaccinated. Global concern over vaccine-preventable diseases has intensified since the COVID-19 pandemic: the Global Burden of Disease 2023 analysis found that immunization-programme disruptions contributed to stagnating or declining vaccine coverage in numerous countries [9], and several countries have since reported renewed measles activity, even in regions where elimination goals had previously been achieved [2,9,25]. Türkiye has long maintained a national immunization programme incorporating two-dose MMR vaccination, with generally high reported coverage, yet recent epidemiological data document renewed measles activity and localised outbreaks, consistent with the waning vaccine-induced immunity and cohort-level susceptibility gaps described across Europe, Asia, and North America [9,13,25,26,27]. Comprehensive, long-term seroepidemiological data on measles, mumps, and rubella immunity among Turkish adults remain limited, particularly regarding temporal changes in seropositivity, age-specific immunity patterns, and the persistence of vaccine-derived protection into adulthood. Therefore, the present study aimed to characterise the seroepidemiology of measles, mumps, and rubella among adults at a tertiary-care university hospital in Istanbul over a nine-year period, with the aim of informing serological surveillance strategies and contributing to international elimination efforts.

## 2. Materials and Methods

### 2.1. Study Design and Setting

This retrospective laboratory-based seroepidemiological study was conducted at the Department of Medical Microbiology, Istanbul University Faculty of Medicine, Istanbul, Türkiye. Laboratory records of measles, rubella, and mumps serological tests performed between January 2014 and December 2022 were retrieved from the hospital laboratory information system (LIS). The aim of this study was to evaluate the seroprevalence patterns of measles, mumps, and rubella antibodies among adults, investigate temporal trends in immunity, identify demographic factors associated with seropositivity, evaluate rubella seroprotection among women of reproductive age, and characterise potential immunity gaps that may inform surveillance strategies.

### 2.2. Study Population

All available measles, rubella, and mumps enzyme-linked immunosorbent assay (ELISA) results performed during the study period were screened, and only adult records (≥18 years at the time of testing) were retained; pediatric patients (<18 years) were excluded. The final study population consisted of adult test records corresponding to six serological assays: Measles IgM, Measles IgG, Rubella IgM, Rubella IgG, Mumps IgM, Mumps IgG. Records for 2014–2019 were exported from the laboratory information system as a single combined dataset spanning that six-year interval, whereas 2020, 2021, and 2022 were each retrieved as separate annual exports; consequently, the 2014–2019 period is presented and analysed as one pooled category throughout this manuscript, while 2020–2022 are presented at annual resolution. This reflects how the source data were made available rather than an analytic choice (rationale in Section 2.4; limitation discussed in Section 4.5). Demographic variables extracted from the database included age, sex, year of testing, and serological test results.

### 2.3. Serological Testing

Serological analyses were performed in the Virology Laboratory of Istanbul University Faculty of Medicine. Serological testing was performed using commercially available ELISA kits manufactured by Vircell S.L. (Granada, Spain): Measles IgM ELISA, Measles IgG ELISA, Rubella IgM ELISA, Rubella IgG ELISA, Mumps IgM ELISA and Mumps IgG ELISA. All assays were performed on the Triturus® automated ELISA processing system (Grifols S.A., Barcelona, Spain) according to the manufacturer’s instructions. Results were interpreted using the manufacturer’s recommended cut-off values and classified as positive, negative, or equivocal, with equivocal results excluded from positivity-rate calculations and inferential analyses. IgG antibodies were used as markers of previous exposure and/or vaccine-induced immunity, whereas IgM antibodies were considered indicators of recent or acute infection.

### 2.4. Data Management

Laboratory records were anonymized before analysis. Where the same individual underwent repeated testing during the study period, all records were retained, because the primary analytical unit of this study was the serological test result rather than the individual patient. This is consistent with the laboratory-based seroepidemiological design, in which serial results reflect real-world testing patterns; it does not bias population-level seropositivity estimates when repeat testing is infrequent and evenly distributed across periods. Age was categorized into four predefined groups:

- 18–29 years
- 30–39 years
- 40–49 years
- ≥50 years

The years 2014–2019 were combined because historical laboratory records for this period were stored as aggregated files rather than individual annual datasets. Annual sample volumes during 2014–2019 were also lower than in subsequent years, and pooling was performed to ensure adequate statistical power for trend comparisons across study periods.

### 2.5. Analysis of Rubella Seroprotection Among Women of Reproductive Age

Because of the public health importance of congenital rubella syndrome (CRS), a subgroup analysis was performed among women of reproductive age (18–49 years). Rubella IgG seroprotection rates were calculated overall and stratified by year and age group. Observed seroprotection levels were compared with the 95% immunity threshold recommended by the World Health Organization (WHO) for rubella elimination; this comparison is used descriptively, as WHO elimination criteria incorporate additional surveillance and vaccination-coverage indicators beyond seroprevalence alone.

### 2.6. Statistical Analysis

Statistical analyses were performed using IBM SPSS Statistics version 29.0 (IBM Corp., Armonk, NY, USA) and R software version 4.3.2 (R Foundation for Statistical Computing, Vienna, Austria). Categorical variables were summarized as frequencies and percentages, whereas continuous variables were expressed as mean (SD), median, and interquartile range (IQR), as appropriate. Seropositivity rates were calculated as the proportion of positive results among all valid positive and negative results, with equivocal results excluded from the denominator. Associations between seropositivity and demographic variables were evaluated using Pearson’s chi-square test. Age-specific analyses were conducted across four predefined age strata (18–29, 30–39, 40–49, and ≥50 years). Temporal changes in seropositivity rates were assessed using the Cochran–Armitage trend test. The direction and strength of monotonic trends were additionally explored using Spearman rank correlation coefficients. The Cochran–Armitage trend test treats the four study periods as ordered categories with equally spaced scores; because the 2014–2019 reference period spans six calendar years whereas 2020, 2021, and 2022 each represent a single year, this and the year-coding used in the regression models below assume equal temporal spacing between periods of unequal length, and the resulting trend statistics should be interpreted as indicating the direction of change rather than a precise linear rate. To assess factors associated with IgG seropositivity, separate multivariable binary logistic regression models were constructed for Measles IgG, Rubella IgG, and Mumps IgG outcomes. For this reason, year was specified as a categorical variable rather than as a linear or ordinal predictor in the primary logistic regression models, with 2014–2019 as the reference category (dummy variables for 2020, 2021, and 2022), alongside age group and sex as covariates. For comparability with studies that model year as a continuous predictor, a supplementary analysis also modelled year as a linear ordinal variable (2014–2019 = 1, 2020 = 2, 2021 = 3, 2022 = 4); results are presented in Supplementary Table S1. Results were reported as odds ratios (ORs) with 95% confidence intervals (CIs). For regression analyses, the youngest age category (18–29 years) and male sex were used as reference groups. Statistical significance was defined as a two-sided *p*-value < 0.05. The *n* = 10,407 records analysed throughout reflect the removal of 54 exact duplicate records during data cleaning (Section 2.2); the effect on seropositivity estimates is discussed in Section 4.5. Given the descriptive, hypothesis-generating nature of this study and the large number of statistical comparisons performed (six trend tests, three age-stratified chi-square tests, six sex-stratified chi-square tests, and three multivariable logistic regression models), no correction for multiple comparisons was applied; *p*-values should therefore be interpreted as indicative rather than strictly confirmatory, particularly for associations close to the *p* = 0.05 threshold (e.g., the Mumps IgG age-group association, *p* = 0.030).

### 2.7. Ethical Approval

The study was approved by the Ethics Committee of Istanbul University, Istanbul Faculty of Medicine (approval number: 2067/29.12.2023, approved on 29 December 2023). Because of the retrospective nature of the study and the use of anonymized laboratory records, the requirement for informed consent was waived by the ethics committee. The study was conducted in accordance with the principles of the Declaration of Helsinki.

## 3. Results

### 3.1. Study Population and Descriptive Statistics

A total of 10,461 adult (≥18 years) ELISA test records were initially retrieved from the laboratory information system, covering the period 2014–2022. During data cleaning, 54 exact duplicate records (identical sample identifier, result, and test date), all in the 2022 Measles IgG dataset, were removed, yielding 10,407 records for analysis. These records corresponded to six distinct assays: Measles IgM, Measles IgG, Rubella IgM, Rubella IgG, Mumps IgM, and Mumps IgG.

The overall mean age of the adult study population was 31.6 (11.1) years (median: 29 years; IQR: 24–36). Female patients constituted 69.6% of the study population (*n* = 7240), reflecting the predominance of women of reproductive age seeking prenatal and gynaecological screening for rubella. The annual distribution ranged from 1052 records in 2020 to 2337 records in 2021. Demographic characteristics stratified by year are presented in Table 1.

### 3.2. IgM and IgG Positivity Rates by Antigen and Year

**Figure 1:**
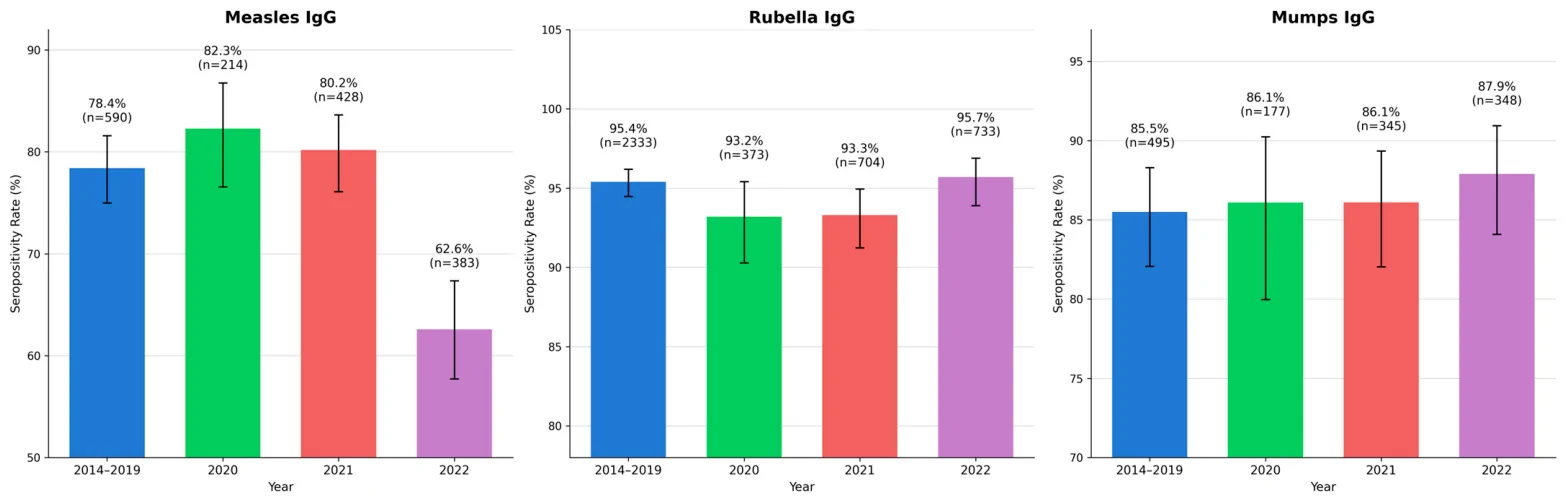
IgG seropositivity rates (%) in adults (≥18 years) by year (2014–2022). Error bars represent 95% Wilson confidence intervals. Measles IgG positivity was significantly lower in 2022 than in earlier periods (Cochran–Armitage trend test, *p* < 0.001).

**Figure 2:**
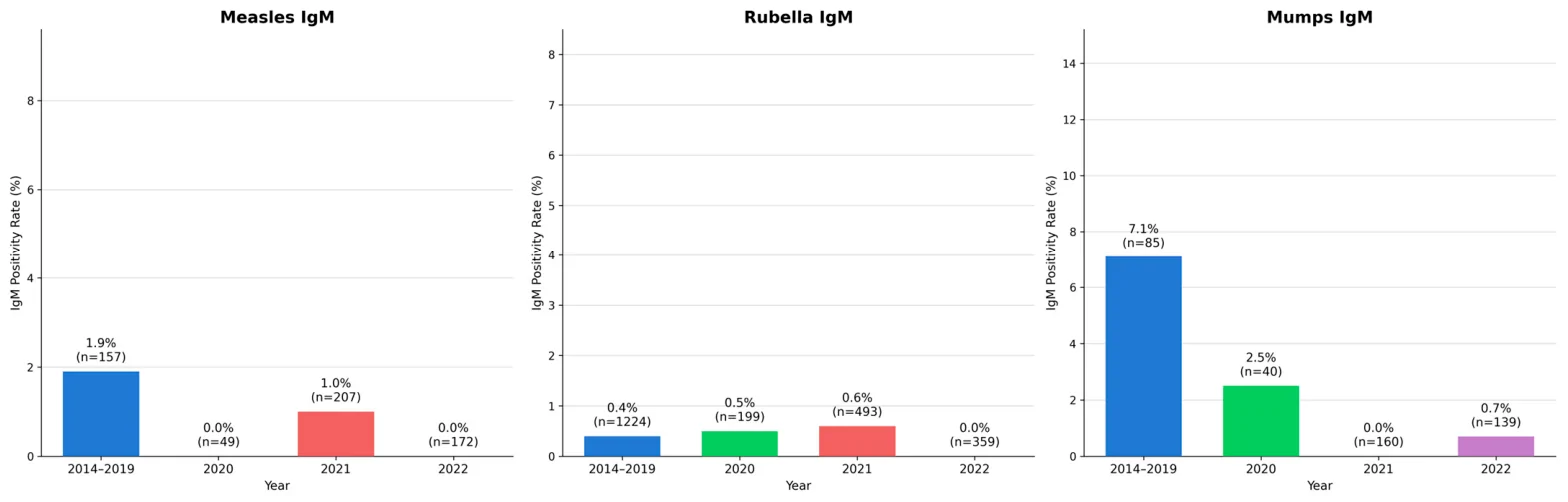
IgM positivity rates (%) in adults (≥18 years) by year. Rates are consistently low, indicating low IgM positivity among adults tested at this institution throughout the study period.

The overall IgG seropositivity rates in the adult population were 75.8% for Measles, 94.9% for Rubella, and 86.3% for Mumps across the entire study period. For IgM (acute infection marker), positivity rates were consistently low: 0.9% for Measles, 0.4% for Rubella, and 1.9% for Mumps. Complete positivity rates stratified by year and test type are presented in Table 2. Temporal trends in IgG are illustrated in Figure 1 and temporal trends in IgM are shown in Figure 2.

### 3.3. Temporal Trend Analysis (Cochran–Armitage Test)

Cochran–Armitage trend tests were applied to detect statistically significant monotonic trends in positivity rates across the four study periods (interpreted subject to the caveats on unequal period length and record independence noted in Section 2.6 and Section 4.5). Two statistically significant trends were identified (Table 3).

Measles IgG seropositivity showed a statistically significant declining pattern across the four study periods (Cochran–Armitage Z = −4.424, *p* < 0.001; Spearman ρ = −0.40): rates were relatively stable from 78.4% in 2014–2019 to 82.3% in 2020 and 80.2% in 2021, before declining sharply to 62.6% in 2022. Mumps IgM positivity also showed a statistically significant decreasing pattern (Cochran–Armitage Z = −3.473, *p* < 0.001; Spearman ρ = −0.80): rates decreased from 7.1% in 2014–2019 to 2.5% in 2020 and 0.0% in 2021, before rising slightly to 0.7% in 2022 (Section 4.2).

In contrast, Rubella IgG seropositivity remained consistently high and stable across all years (93.2–95.7%; Z = −0.631, *p* = 0.528). Mumps IgG showed a non-significant upward trend (85.5% to 87.9%; Z = 0.916, *p* = 0.360), and both Measles IgM and Rubella IgM showed no significant temporal trend (*p* > 0.05). These results are summarised in Table 3.

### 3.4. Age Group Stratification and Immunity Gap Analysis

**Figure 3:**
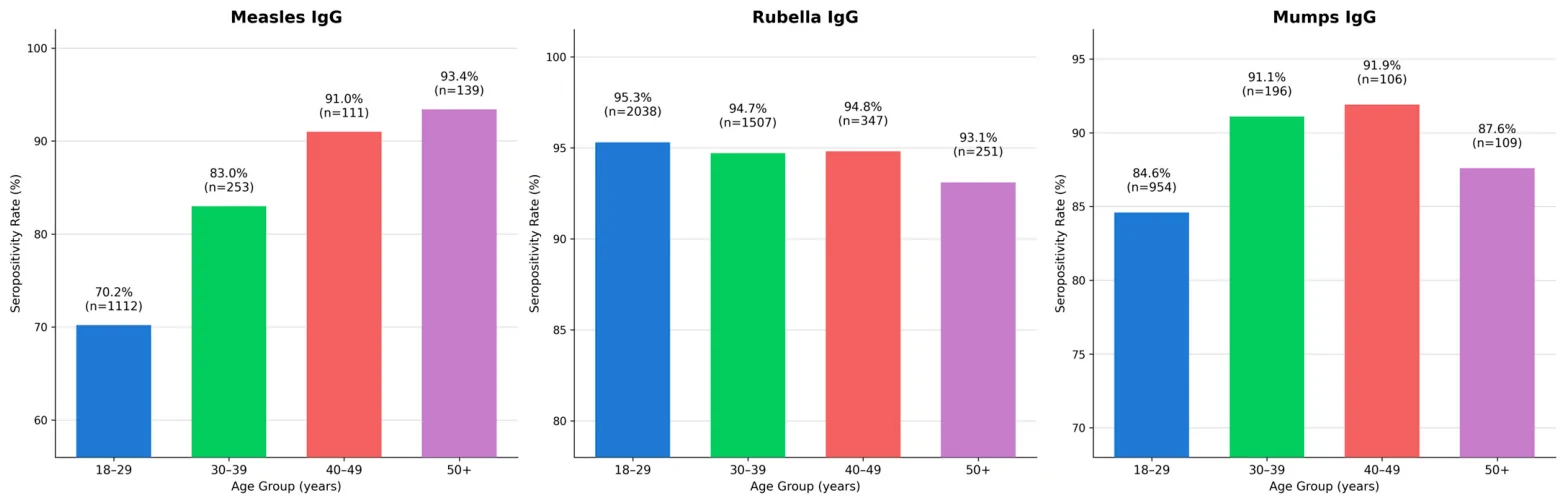
IgG seropositivity rates (%) by adult age group (18–29, 30–39, 40–49, ≥50 years). Measles and Mumps IgG show significant age-stratified variation (*p* < 0.05), with younger adults displaying lower seropositivity.

To assess the relationship between age group and IgG seropositivity—a proxy for vaccine-induced immunity gaps—chi-square tests of independence were conducted across four adult age strata (18–29, 30–39, 40–49, and ≥50 years) for each IgG test. Results are presented in Table 4 and Figure 3.

For Measles IgG, a statistically significant and clinically meaningful association was observed between age group and seropositivity (χ^2^ = 62.02, df = 3, *p* < 0.001). Seropositivity increased monotonically with age, from 70.2% (18–29 years) to 83.0% (30–39), 91.0% (40–49), and 93.4% (≥50 years) (interpreted further in Section 4.1).

For Mumps IgG, a nominally significant association was also found (χ^2^ = 8.95, df = 3, *p* = 0.030; interpreted cautiously given the absence of correction for multiple comparisons, Section 2.6), with seropositivity rising from 84.6% in the youngest group to 91.9% at 40–49 years before declining modestly to 87.6% at ≥50 years (Section 4.2).

Rubella IgG seropositivity showed no statistically significant variation across age groups (χ^2^ = 2.25, df = 3, *p* = 0.523), with uniformly high rates across all strata (93.1–95.3%), suggesting robust rubella immunity across all ages.

### 3.5. Gender Differences in Seropositivity

Seropositivity rates were compared between adult male and female patients using chi-square tests for all six assays (Table 5). No statistically significant gender-based differences were detected for any of the six tests (all *p* > 0.05): Measles IgG was 76.5% in males vs. 75.1% in females (χ^2^ = 0.35, *p* = 0.556), Rubella IgG was 95.5% vs. 94.7% (χ^2^ = 0.73, *p* = 0.393), and Mumps IgG was 86.2% vs. 86.5% (χ^2^ = 0.01, *p* = 0.935). Sex therefore does not appear to be an independent determinant of MMR seropositivity in this hospital-based population—though, since the female predominance here (69.6%) reflects rubella prenatal-screening referrals rather than random sampling, pooling the comparison across all six assays without stratifying by testing indication could mask assay-specific differences, and this null result should not be read as evidence of no sex-related difference in the untested general population (Section 4.5).

### 3.6. Rubella IgG Seroprotection in Women of Reproductive Age

Given the public health implications of rubella susceptibility for Congenital Rubella Syndrome (CRS) prevention, a sub-analysis was conducted for the 3123 Rubella IgG tests performed in women aged 18–49 years during 2014–2022. The overall seroprotection rate was 94.7% (*n* = 2959 positive/3123 total; 95% Wilson CI: 93.9-95.5%). Although the point estimate exceeds the 90% threshold typically required to prevent CRS outbreaks and is close to the 95% population immunity benchmark used in rubella elimination frameworks, its confidence interval spans this benchmark, so this sub-group estimate cannot be considered statistically distinguishable from 95% on its own [7,23]. This is a descriptive benchmark rather than a formal elimination assessment, which would additionally require vaccination-coverage and surveillance data beyond seroprevalence alone.

**Figure 4:**
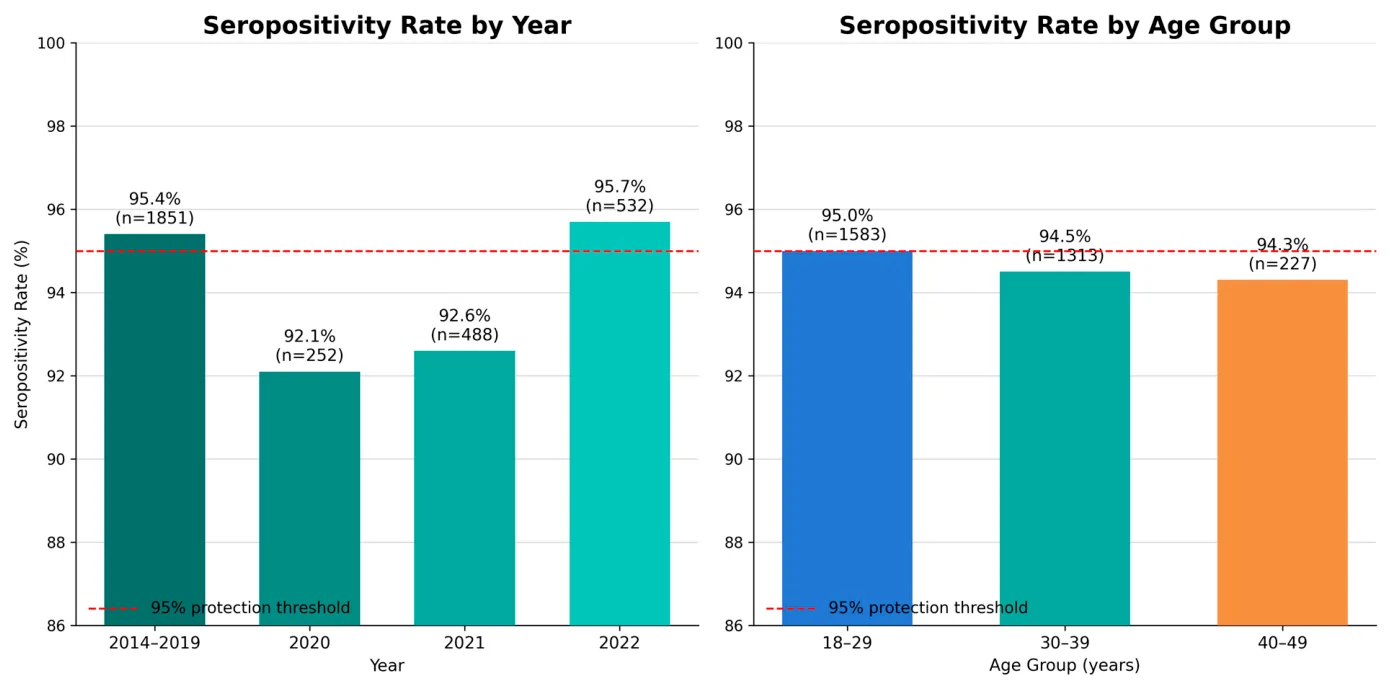
Rubella IgG seroprotection rates in women of reproductive age (18–49 years) by year (left panel) and by age group (right panel). The dashed red line indicates the 95% WHO seroprevalence benchmark for rubella elimination. All strata approach but do not consistently exceed this benchmark.

By year, seroprotection rates were 95.4% in 2014–2019, 92.1% in 2020, 92.6% in 2021, and 95.7% in 2022. By age group, the 18–29 year stratum showed the highest IgG positivity (95.0%), followed by 30–39 years (94.5%) and 40–49 years (94.3%). These data are visualised in Figure 4.

### 3.7. Multivariate Logistic Regression Analysis

**Figure 5:**
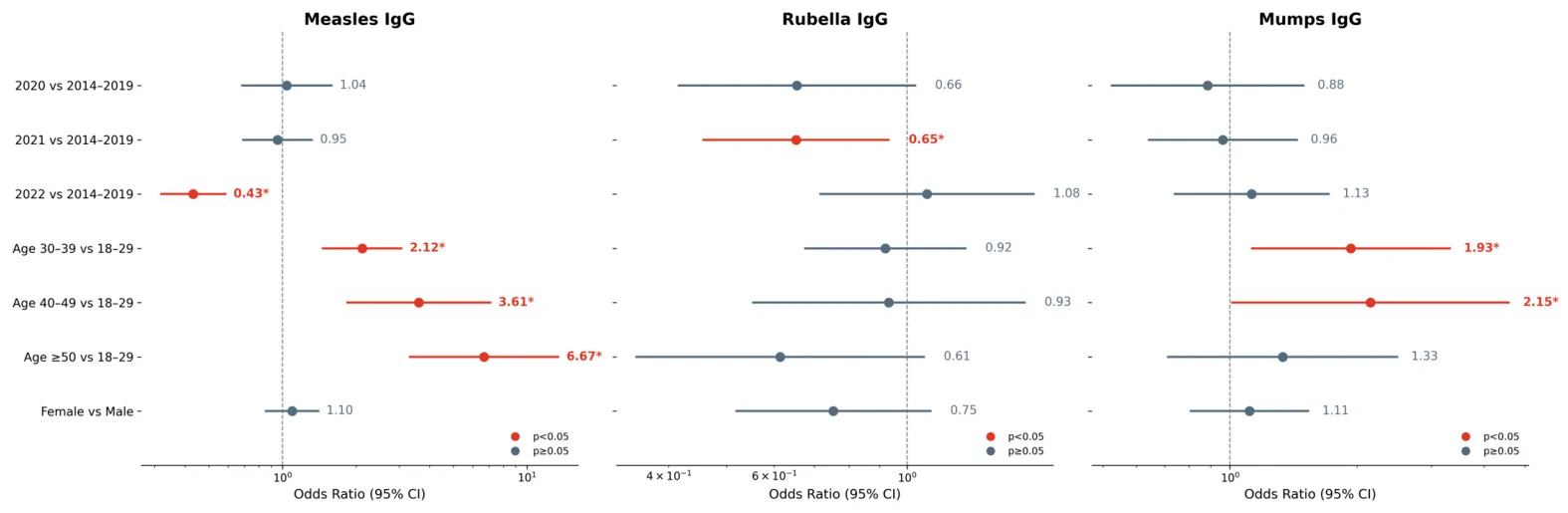
Forest plot of odds ratios (OR) from multivariate logistic regression models for Measles, Rubella, and Mumps IgG seropositivity. Red markers and asterisks (*) indicate statistically significant associations (*p* < 0.05). All models adjusted for year (categorical; reference: 2014–2019), age group, and sex. Log scale on *x*-axis.

Multivariate binary logistic regression models were constructed for each IgG outcome (Measles, Rubella, Mumps) to explore associations with seropositivity, adjusted for year (categorical; reference: 2014–2019), age group (four categories, referent: 18–29 years), and sex (referent: male). These analyses should be considered exploratory given the absence of vaccination records and other important confounders. Odds ratios (OR) with 95% confidence intervals are presented in Table 6 and visualised as a forest plot in Figure 5. A sensitivity analysis retaining year as a linear ordinal variable yielded substantively concordant results (Supplementary Table S1). For Measles IgG, a supplementary age × year interaction model is presented in Table 7.

For Measles IgG, the categorical year model identified 2022 as the only period with significantly lower seropositivity relative to 2014–2019 (OR = 0.432, 95% CI: 0.319–0.584, *p* < 0.001), whereas 2020 (OR = 1.041, *p* = 0.851) and 2021 (OR = 0.955, *p* = 0.779) did not differ significantly from baseline, localising the decline to the 2022 testing period. Older age groups remained strong independent positive predictors: compared to the 18–29 year reference, the OR for seropositivity was 2.12 (95% CI: 1.47–3.06) for the 30–39 year group, 3.61 (95% CI: 1.84–7.07) for the 40–49 year group, and 6.67 (95% CI: 3.32–13.41) for those aged ≥50 years (all *p* < 0.001). Sex was not a significant predictor (OR = 1.10, *p* = 0.469; Section 3.5).

For Rubella IgG, the only significant association identified in the multivariable model was for the 2021 period, which showed significantly lower odds of seropositivity relative to 2014–2019 (OR = 0.653, 95% CI: 0.457–0.932, *p* = 0.019); 2020 (OR = 0.655, 95% CI: 0.416–1.032, *p* = 0.068) and 2022 (OR = 1.080, 95% CI: 0.717–1.626, *p* = 0.714) did not differ significantly from baseline. Neither sex (female vs. male: OR = 0.753, 95% CI: 0.519–1.094, *p* = 0.137) nor age group, including the ≥50-year stratum (OR = 0.614, 95% CI: 0.353–1.067, *p* = 0.084), was an independent predictor of Rubella IgG seropositivity in the adjusted model (all *p* > 0.05).

For Mumps IgG, older age groups showed higher odds of seropositivity in the 30–39 (OR = 1.93, 95% CI: 1.13–3.31, *p* = 0.017) and 40–49 (OR = 2.15, 95% CI: 1.01–4.57, *p* = 0.046) year groups compared to the youngest adults, a pattern consistent with a greater cumulative likelihood of natural infection history in older cohorts; the 40–49 year contrast should be interpreted cautiously given its borderline *p*-value and wide confidence interval. None of the year contrasts were significant for Mumps IgG in the categorical model (2020 vs. 2014–2019: OR = 0.885, *p* = 0.646; 2021 vs. 2014–2019: OR = 0.962, *p* = 0.849; 2022 vs. 2014–2019: OR = 1.126, *p* = 0.579).

To formally test whether the temporal decline in Measles IgG seropositivity differed by age, an additional logistic regression model was fitted with a period × age (18–29 vs. ≥30 years) interaction term, adjusted for sex. The interaction was significant overall (likelihood-ratio χ^2^ = 10.75, df = 3, *p* = 0.013; Table 7): the odds of seropositivity among 18–29-year-olds declined significantly more than among adults aged ≥30 years during 2021 (interaction OR = 0.297, 95% CI: 0.129–0.685, *p* = 0.004) and 2022 (interaction OR = 0.357, 95% CI: 0.166–0.771, *p* = 0.009), while the 2020 term was not significant (OR = 0.466, 95% CI: 0.180–1.205, *p* = 0.115). Because age group is closely intertwined with birth cohort in a cross-sectional design, this is best described as an age-group-specific temporal pattern rather than direct evidence of a causal age effect. The raw rates make the pattern concrete: among adults aged ≥30 years, Measles IgG seropositivity remained stable or even rose across the study period (83.2% in 2014–2019, 90.5% in 2020, 92.9% in 2021, and 83.5% in 2022), while among those aged 18–29 it declined progressively (76.8%, 75.0%, 72.1%, and 54.6%, respectively). The 2022 decline in this hospital-tested population is therefore driven predominantly by the youngest adult stratum rather than a uniform loss across all age groups.

## 4. Discussion

This nine-year retrospective seroepidemiological study of measles, mumps, and rubella immunity among adults at a tertiary-care university hospital in Istanbul points to three principal findings. Measles IgG seropositivity declined significantly over the study period, falling from 78.4% in 2014–2019 to 62.6% in 2022, with the steepest deficit concentrated in adults aged 18–29 years, while Rubella IgG remained consistently high and stable, though seroprotection among women of reproductive age did not consistently reach the 95% WHO seroprevalence benchmark for rubella elimination. Mumps IgG was likewise stable across the observation window, whereas Mumps IgM positivity decreased markedly during the COVID-19 pandemic period, suggesting a possible reduction in acute viral transmission while non-pharmaceutical interventions were widely implemented.

### 4.1. Measles IgG Seropositivity and Temporal Trends

The observed decline in measles seropositivity is consistent with a cohort-based immunity gap: younger adults in our study cohort were born during or after the introduction of routine MMR vaccination in Türkiye and depend predominantly on two-dose vaccine-derived immunity, whereas older adults (aged ≥40 years) are more likely to carry naturally acquired immunity through prior exposure to circulating wild-type measles virus. This echoes recent seroepidemiological reports of reduced measles antibody persistence in vaccine-era cohorts elsewhere, particularly since the COVID-19 pandemic [6,10,28,29]; Equils et al. further note that reduced vaccine effectiveness may reflect not only waning host immunity but also primary and secondary vaccine failures, particularly in immunocompromised individuals and those with chronic conditions [29]. For context, Quach et al. reported measles seropositivity of only 53.4% among adults aged 20–44 years in a highly MMR-vaccinated US population [30]; Centrone et al. found susceptibility ranging from 39.8% (blood donors aged 18–24) to 4.0% (aged 45–54) in southern Italy (overall seroprevalence 85.3%, 95% CI: 83.4–87.0) [11]; and Friedrich et al. reported 14.5% seronegativity (95% CI: 11.6–17.9%) in the youngest German birth cohort (1985–1993) [18]. Similar susceptibility has been reported among healthcare workers in Madrid [31] and among vaccinated populations in Hungary, Croatia, and South Korea [32,33].

The relatively low measles seropositivity observed in the youngest adult cohort deserves particular attention. Our cross-sectional design cannot directly distinguish between true within-individual immunological waning and cohort-related differences in exposure history, but several mechanisms plausibly contribute to it. Individuals aged 18–29 years depend predominantly on two-dose vaccine-induced immunity, which carries its own baseline of seronegativity: primary vaccine failure occurs in roughly 2–5% of recipients [29], and secondary vaccine failure—gradual antibody waning years to decades after seroconversion—is increasingly recognised as a further contributor among vaccine-era adults [10,29]. Because vaccination records, prior infection history, and immune status were unavailable, this analysis cannot distinguish these mechanisms from other possible explanations (incomplete vaccination, non-response, seroreversion after infection, or immunosuppression); the estimates should be read as a population-level snapshot rather than attribution to any single cause. Seropositivity among individuals aged 18–29 years was 70.2% overall, and fell to 54.6% in 2022, a pattern that mirrors the accumulation of susceptible young adults reported in comparable vaccine-era cohorts elsewhere [11,18,30,34].

The consistently higher Measles IgG seropositivity among older adults is best explained by cohort effects rather than age-related immune enhancement: this group is more likely to have acquired natural immunity through exposure to circulating wild-type virus before routine MMR vaccination began in Türkiye. Kurita et al. reached a similar conclusion for measles, rubella, mumps, and varicella in Japanese adults, whose pre-vaccine-era cohorts retained substantially higher and more durable antibody levels than those relying solely on vaccination [24]; Grassi et al. reported the same pattern—lowest seroprevalence in young adults—in the Italian general population in 2019–2020 [34], and a comparably graded, birth-cohort-stratified pattern spanning birth years 1922–2024 has been reported in Austria [35]. Our regression results support this reading directly, with the odds of measles seropositivity rising with each successive age group to an OR of 6.67 (95% CI 3.32–13.41) for adults aged ≥50 relative to those aged 18–29.

The temporal decline in measles seropositivity observed between 2020 and 2022 may have been compounded by pandemic-related disruptions. No changes in assay platform, cut-off values, or laboratory workflow were recorded during the study period, though this was not independently audited against lot-level quality-control data, and shifts in referral patterns or testing indications cannot be fully excluded in this retrospective dataset. Critically, the decline was not uniform across age strata: the age × year interaction confirmed it was statistically concentrated among adults aged 18–29 years (Section 3.7, Table 7), which argues against a hospital-wide artefact, since a change in assay lot or referral pathway would be expected to affect all ages similarly. A modest shift in the basic demographic composition of the tested Measles IgG population was observed in 2022 relative to 2020–2021 (mean age 30.2 years, 54.7% female in 2022, versus 32.1 years/48.6% female in 2020 and 31.9 years/51.9% female in 2021); because age and sex are already covariates in the regression model, this shift alone does not explain the adjusted 2022 effect, though the absence of any recorded referral source or clinical indication in the laboratory data means a testing-indication change specific to 2022 cannot be ruled out. This coincides with well-documented pandemic-era disruption to routine immunisation services worldwide: Ota et al. reported widespread disruption to childhood vaccination programmes, with downstream concerns about measles resurgence [36]. Globally, an estimated 25 million children missed their first measles vaccine dose in 2021 alone—the highest number in over a decade [26]. The pronounced 2022 decline should therefore be read cautiously, as a likely mix of true population-level immunity change and shifts in healthcare utilisation or testing indications that this retrospective design cannot fully disentangle. Wang et al. specifically quantified how pandemic-era reductions in both vaccination and natural virus circulation created compound immunity deficits in the measles-susceptible pediatric and young adult population [28]. Recent reports from Türkiye have also documented renewed measles activity, supporting the epidemiological relevance of the declining seropositivity observed in the present study [13,25].

### 4.2. Mumps IgG and IgM Seropositivity

Regarding mumps, the absence of a significant temporal trend in IgG seropositivity (Section 3.3) is reassuring but should not be read as evidence of complete protection: Yang et al. highlighted waning vaccine-induced mumps immunity as a recognised contributor to outbreaks in highly vaccinated populations, with boosting from natural exposure progressively lost as vaccine-era cohorts age [6], and Risalvato similarly found that antibody levels can decline years after vaccination, with booster doses producing seroconversion of uncertain durability [19]. The significant decline in Mumps IgM positivity from 7.1% to 0.0% (2014–2019 to 2021) may partly reflect COVID-19-related social distancing reducing respiratory virus transmission rather than a true long-term change—a pattern described for other droplet-transmitted pathogens, though this retrospective design cannot establish that mechanism directly; no significant sex-related differences were identified. The significant age-group association for Mumps IgG (χ^2^ = 8.95, *p* = 0.030; Section 3.4), corroborated by the adjusted model (Section 3.7), parallels the pattern seen for Measles IgG and fits Kurita et al.’s interpretation for measles, mumps, rubella, and varicella in Japanese adults, that cohorts exposed to pre-vaccine-era wild-type circulation retain more durable antibody levels than those relying on vaccination alone [24]. Given the absence of correction for multiple comparisons (Section 2.6) and the cross-sectional design, this is best read as hypothesis-generating rather than definitive evidence of a mumps-specific immunity gap among younger adults.

### 4.3. Rubella IgG Seropositivity and Seroprotection in Women of Reproductive Age

Rubella IgG seropositivity remained stable and high throughout the study period (range 93.2–95.7%, overall 94.9%), with little variation across age groups—consistent with effective implementation of the national MMR programme. Although the unadjusted Cochran–Armitage trend test found no significant temporal trend in Rubella IgG seropositivity (Z = −0.631, *p* = 0.528; Table 3), the adjusted multivariate model identified significantly lower odds of seropositivity in 2021 relative to 2014–2019 (OR = 0.653, 95% CI: 0.457–0.932, *p* = 0.019), with 2020 and 2022 not differing from baseline (Table 6). This discrepancy may reflect confounding by the age or sex composition of the 2021 sample rather than a true additional decline, especially since the raw 2021 rate (93.3%) sits within the study’s overall stable range (93.2–95.7%; Table 2); as one of several period contrasts tested without correction for multiple comparisons, it should be read cautiously and does not on its own suggest an emerging threat to rubella population immunity. Seroprotection among women of reproductive age nonetheless remained slightly below the WHO elimination target, consistent with other vaccinated populations and supporting continued screening of women before pregnancy [7,12,23]; the declining measles immunity observed among young adults in our study population extends this concern beyond individual susceptibility, since waning maternal measles antibody levels are known to widen the window of infant susceptibility before the first MMR dose [17].

### 4.4. IgM Positivity and Acute Infection

Consistently low IgM positivity rates for all three antigens throughout the study period (<2% for measles, <2% for mumps, and <1% for rubella) do not provide evidence of active outbreak activity among the adult population presenting to this institution during the study window, although passive laboratory-based surveillance of this kind cannot exclude undetected transmission. The near-zero IgM rates during 2020 and 2021 may reflect both a genuine reduction in transmission due to pandemic-related restrictions and a possible reduction in healthcare-seeking behaviour among mildly symptomatic patients during that period. India-based seroprevalence data from Prosperi et al. using residual blood samples demonstrated similarly low IgM rates in adult populations outside outbreak periods, consistent with IgM positivity generally reflecting acute or very recent infection, although this external comparison does not itself validate the local assay or sampling process used in the present study [37].

### 4.5. Limitations and Strengths

Because the study was conducted at a single tertiary-care hospital, testing indications—prenatal rubella screening, occupational checks, clinical suspicion, and immunosuppression work-up—were non-random, so the seropositivity estimates reflect adults who sought testing here rather than the untested general population; the marked female predominance (69.6%) largely reflects the volume of prenatal Rubella IgG screening and should be kept in mind when interpreting the sex-stratified results.

A reliable patient-level identifier was unavailable for most of the dataset, limiting how confidently records can be treated as independent observations. A numeric identifier existed only for the 2014–2019 Measles and Mumps files (12.8% of records; 745 unique patients, none tested twice for the same analyte), and the 2014–2019 Rubella files (34.2%) could only be linked by name, where a modest number of genuine repeats (53 of 2276 unique names in the IgG file, 31 of 1191 in the IgM file) meant that restricting to one record per name shifted seropositivity by no more than 0.1 percentage points. For the remaining 53.1% of records (2020–2022), only an anonymised sample-level code was available, so neither linkage nor a patient-clustered model (e.g., GEE or mixed-effects logistic regression) was possible; the *p*-values and confidence intervals reported throughout therefore assume independent records, an assumption unverifiable for most of the sample.

Separately, data cleaning removed 54 exact duplicate records—all in the 2022 Measles IgG dataset—before analysis (*n* = 10,407 reported throughout); because these duplicates were disproportionately positive, removing them left the pooled Measles IgG rate essentially unchanged but pulled the 2022-specific rate down from 64.3% to 62.6% (Rubella and Mumps IgG were unaffected). Finally, the 2014–2019 period appears as a single pooled category because it was exported from the laboratory system as one combined file, whereas 2020–2022 are available at annual resolution—a coarser temporal grain for the earliest years.

## 5. Conclusions

This nine-year retrospective seroepidemiological study identified a significant temporal decline in Measles IgG seropositivity among adults in Istanbul, falling from 78.4% in 2014–2019 to 62.6% in 2022 (*p* < 0.001), with the most pronounced immunity gap in young adults aged 18–29 years (70.2% overall). Although the cross-sectional design precludes attributing this to within-individual antibody waning, the pattern is consistent with a cohort effect in which vaccine-era adults show lower seropositivity than older adults with naturally acquired immunity. Rubella IgG remained consistently high (93.2–95.7%) and Mumps IgG was stable (85.5–87.9%) throughout the study period. Rubella seroprotection among women of reproductive age was 94.7% (95% CI: 93.9–95.5%), close to but not statistically distinguishable from the 95% population immunity benchmark used in rubella elimination frameworks [7,23]. Nonetheless, several strata—by calendar year and by age group—fell just below this benchmark. Measles and Mumps IgM positivity remained low throughout (<2%), consistent with low acute infection activity at this institution. These findings point to several public health implications: (i) targeted booster vaccination for adults aged 18–29 years—the age band where the 2022 decline was concentrated (Table 7)—rather than vaccine-era adults broadly; (ii) continued rubella screening before and during pregnancy, with catch-up MMR vaccination for women found seronegative ahead of a future pregnancy; (iii) ongoing standardised serological surveillance to monitor population immunity and guide vaccination policy in Türkiye; (iv) a population-representative, non-hospital-referred seroprevalence study confirming whether this age-related susceptibility gap extends beyond the tested clinical population; and (v) adoption of a consistent patient-level identifier across assays and reporting periods in the laboratory information system, enabling robust longitudinal and clustering-adjusted analyses in future work. Key limitations include the hospital-based retrospective design, absence of individual vaccination records, and the pooled structure of 2014–2019 data. Future prospective studies incorporating vaccination history, longitudinal follow-up, and community-based sampling are needed to characterise the long-term persistence of MMR immunity and optimise booster strategies for Turkish adults.

## Figures and Tables

**Table 1:** Demographic characteristics of the adult study population by year (2014–2022).

Year	*n* (Adults)	Age Mean (SD)	Age Median (IQR)	Female *n* (%)	Male *n* (%)
2014–2019	4884	30.5 (9.8)	28 (23–34)	3673 (75.2%)	1211 (24.8%)
2020	1052	33.2 (11.6)	30 (25–38)	681 (64.7%)	371 (35.3%)
2021	2337	32.6 (11.7)	29 (24–38)	1492 (63.8%)	845 (36.2%)
2022	2134	32.0 (12.5)	28 (24–36)	1394 (65.3%)	740 (34.7%)
Total	10,407	31.6 (11.1)	29 (24–36)	7240 (69.6%)	3167 (30.4%)

IQR: Interquartile Range.

**Table 2:** IgM and IgG positivity rates in adults (≥18 years) by antigen and year.

Test	2014–2019 *n*	2014–2019%	2020 *n*	2020%	2021 *n*	2021%	2022 *n*	2022%
Measles IgM	157	1.9	49	0.0	207	1.0	172	0.0
Rubella IgM	1224	0.4	199	0.5	493	0.6	359	0.0
Mumps IgM	85	7.1	40	2.5	160	0.0	139	0.7
Measles IgG	590	78.4	214	82.3	428	80.2	383	62.6
Rubella IgG	2333	95.4	373	93.2	704	93.3	733	95.7
Mumps IgG	495	85.5	177	86.1	345	86.1	348	87.9

*n*: number of tests performed. %: percentage of IgM- or IgG-positive results among tests with valid categorical result (Positive/Negative/Equivocal). Equivocal results were excluded from positivity rate calculations.

**Table 3:** Cochran–Armitage trend test results for IgM and IgG positivity rates across study periods.

Test	Trend Direction	Z Statistic	*p*-Value	Significant
Measles IgM	Decreasing	−1.692	0.091	No
Rubella IgM	Stable	−0.557	0.578	No
Mumps IgM	Decreasing	−3.473	<0.001	Yes *
Measles IgG	Decreasing	−4.424	<0.001	Yes *
Rubella IgG	Stable	−0.631	0.528	No
Mumps IgG	Increasing	0.916	0.360	No

* Statistically significant at *p* < 0.05. Z: Cochran–Armitage Z statistic. ρ: Spearman rank correlation coefficient for direction assessment. Trend tests assign equally spaced scores to the four study periods despite their unequal calendar length (see Section 2.6).

**Table 4:** Chi-square analysis of IgG seropositivity rates across adult age groups (18–29, 30–39, 40–49, ≥50 years).

Test	18–29 yr %	30–39 yr %	40–49 yr %	≥50 yr %	χ^2^ (df = 3)	*p*-Value
Measles IgG	70.2	83.0	91.0	93.4	62.02	<0.001 *
Rubella IgG	95.3	94.7	94.8	93.1	2.25	0.523
Mumps IgG	84.6	91.1	91.9	87.6	8.95	0.030 *

* *p* < 0.05. χ^2^: Pearson chi-square statistic. df: degrees of freedom. All positivity rates exclude equivocal results.

**Table 5:** Chi-square analysis of MMR seropositivity rates by gender in the adult study population.

Test	Male Positive %	Female Positive %	χ^2^	*p*-Value
Measles IgM	0.4	1.2	0.456	0.499
Rubella IgM	0.0	0.5	0.926	0.336
Mumps IgM	2.0	1.8	0.000	1.000
Measles IgG	76.5	75.1	0.347	0.556
Rubella IgG	95.5	94.7	0.729	0.393
Mumps IgG	86.2	86.5	0.007	0.935

All *p*-values > 0.05 (non-significant). All six assays include the full 2014–2022 study period.

**Table 6:** Multivariate logistic regression: odds ratios (OR) and 95% confidence intervals for IgG seropositivity in adults (≥18 years).

Test/Variable	Odds Ratio	95% CI Lower	95% CI Upper	*p*-Value
**Measles IgG**				
2020 vs. 2014–2019	1.041	0.683	1.587	0.851
2021 vs. 2014–2019	0.955	0.691	1.319	0.779
2022 vs. 2014–2019	0.432	0.319	0.584	<0.001 *
Age 30–39 vs. 18–29	2.118	1.465	3.061	<0.001 *
Age 40–49 vs. 18–29	3.611	1.843	7.074	<0.001 *
Age ≥ 50 vs. 18–29	6.675	3.322	13.412	<0.001 *
Female vs. Male	1.095	0.856	1.401	0.469
**Rubella IgG**				
2020 vs. 2014–2019	0.655	0.416	1.032	0.068
2021 vs. 2014–2019	0.653	0.457	0.932	0.019 *
2022 vs. 2014–2019	1.080	0.717	1.626	0.714
Age 30–39 vs. 18–29	0.920	0.676	1.252	0.596
Age 40–49 vs. 18–29	0.932	0.553	1.571	0.793
Age ≥ 50 vs. 18–29	0.614	0.353	1.067	0.084
Female vs. Male	0.753	0.519	1.094	0.137
**Mumps IgG**				
2020 vs. 2014–2019	0.885	0.525	1.492	0.646
2021 vs. 2014–2019	0.962	0.643	1.439	0.849
2022 vs. 2014–2019	1.126	0.741	1.711	0.579
Age 30–39 vs. 18–29	1.933	1.127	3.315	0.017 *
Age 40–49 vs. 18–29	2.151	1.012	4.571	0.046 *
Age ≥50 vs. 18–29	1.333	0.714	2.488	0.367
Female vs. Male	1.112	0.808	1.532	0.514

* *p* < 0.05. Reference categories: Age 18–29 years; Male sex; Year 2014–2019. OR: Odds Ratio. CI: Confidence Interval. Year modelled as categorical variable (reference: 2014–2019; dummy variables for 2020, 2021, and 2022). The supplementary linear ordinal model results are presented in Supplementary Table S1.

**Table 7:** Measles IgG: logistic regression with a period × age (18–29 vs. ≥30 years) interaction term, adjusted for sex.

Term	Odds Ratio	95% CI Lower	95% CI Upper	*p*-Value
2020 vs. 2014–2019 (age ≥30 reference)	1.949	0.862	4.406	0.109
2021 vs. 2014–2019 (age ≥30 reference)	2.638	1.241	5.608	0.012 *
2022 vs. 2014–2019 (age ≥30 reference)	1.020	0.510	2.040	0.955
Age 18–29 vs. ≥30 (at 2014–2019 baseline)	0.659	0.403	1.079	0.097
Female vs. Male	1.078	0.842	1.380	0.551
2020 × Age 18–29 (interaction)	0.466	0.180	1.205	0.115
2021 × Age 18–29 (interaction)	0.297	0.129	0.685	0.004 *
2022 × Age 18–29 (interaction)	0.357	0.166	0.771	0.009 *

* *p* < 0.05. Model: y ~ period × age_18–29 + sex (*n* = 1531). Likelihood-ratio test for the interaction as a whole: χ^2^ = 10.75, df = 3, *p* = 0.013. This model is a targeted follow-up to Table 6 (which reports the four-level age-group main-effects model) and is restricted to Measles IgG, the only outcome for which Section 3.4 and the Discussion identify an age-related pattern warranting formal testing. Age was dichotomised (18–29 vs. ≥30 years) for this interaction model because the full period × four-level age-group interaction exhibited quasi-complete separation in several sparse strata and did not converge reliably; the binary specification provided a stable and directly interpretable test of whether the temporal trend differs specifically for the youngest adults.

## Data Availability

The datasets generated and/or analyzed during the current study are not publicly available because they contain patient-related data but are available from the corresponding author on reasonable request, subject to institutional and ethical approval.

## Supplementary Materials

The following supporting information can be downloaded at https://www.mdpi.com/article/10.3390/medicina62091709/s1: Table S1. Sensitivity analysis: multivariate logistic regression for IgG seropositivity with year modelled as a linear ordinal variable (2014–2019 = 1, 2020 = 2, 2021 = 3, 2022 = 4, i.e., per successive study period rather than per calendar year) instead of the categorical year variable used in the primary model (Table 6).

## Abbreviations

The following abbreviations are used in this manuscript:

CI	Confidence interval
CRS	Congenital Rubella Syndrome
ELISA	Enzyme-Linked Immunosorbent Assay
IQR	Interquartile Range
IgG	Immunoglobulin G
IgM	Immunoglobulin M
LIS	Laboratory Information System
MMR	Measles–Mumps–Rubella
OR	Odds Ratio
SD	Standard Deviation
WHO	World Health Organization
