# Supplementary material for: Waning Measles Immunity and Sustained Rubella Seroprotection Among Adults in Istanbul, Türkiye
Source: Medicina (Kaunas). 2026 Sep 6;62(9):1709. doi: 10.3390/medicina62091709 (PMC13609437; doi:10.3390/medicina62091709)
Supplement: Supplementary file 1 [file medicina-62-01709-s001.zip › medicina-4439173-supplementary.pdf]

Supplementary Materials

The following supplementary results are referenced in Section 2.6 (Statistical Analysis), Section 3.7 (Multivariate Logistic Regression Analysis), and Section 4.5 (Limitations and Strengths).

Table S1. Sensitivity analysis: multivariate logistic regression for IgG seropositivity with year modelled as a linear ordinal variable (2014–2019 = 1, 2020 = 2, 2021 = 3, 2022 = 4, i.e., per successive study period rather than per calendar year) instead of the categorical year variable used in the primary model (Table 6).

Test / Variable	Odds Ratio	95% CI Lower	95% CI Upper	p-value
**Measles IgG**				
Year (per period, linear)	0.782	0.708	0.864	<0.001 *
Age 30–39 vs 18–29	2.168	1.505	3.125	<0.001 *
Age 40–49 vs 18–29	4.174	2.142	8.133	<0.001 *
Age ≥50 vs 18–29	6.942	3.465	13.909	<0.001 *
Female vs Male	1.088	0.852	1.391	0.499
**Rubella IgG**				
Year (per period, linear)	0.961	0.857	1.078	0.497
Age 30–39 vs 18–29	0.909	0.669	1.237	0.545
Age 40–49 vs 18–29	0.880	0.524	1.479	0.630
Age ≥50 vs 18–29	0.620	0.358	1.076	0.090
Female vs Male	0.785	0.542	1.138	0.202
**Mumps IgG**				
Year (per period, linear)	1.032	0.906	1.175	0.634
Age 30–39 vs 18–29	1.887	1.106	3.218	0.020 *
Age 40–49 vs 18–29	2.089	0.988	4.415	0.054
Age ≥50 vs 18–29	1.307	0.703	2.430	0.397
Female vs Male	1.111	0.807	1.529	0.520

* $p < 0.05$. Reference categories: Age 18–29 years; Male sex. Year modelled as a linear ordinal variable (1 = 2014–2019 … 4 = 2022), i.e. the OR represents the change in odds of IgG seropositivity per successive study period. Compare with the categorical year model in Table 6. n = 1,531 (Measles IgG), 4,111 (Rubella IgG), 1,330 (Mumps IgG), identical to the primary models in Table 6.
